# Pre-linguistic infants employ complex communicative loops to engage mothers in social exchanges and repair interaction ruptures

**DOI:** 10.1098/rsos.170274

**Published:** 2018-01-24

**Authors:** Nadège Bourvis, Magi Singer, Catherine Saint Georges, Nicolas Bodeau, Mohamed Chetouani, David Cohen, Ruth Feldman

**Affiliations:** 1 Service de Psychiatrie de l'Enfant et de l'Adolescent, Groupe Hospitalier Pitié-Salpêtrière, Assistance Publique-Hôpitaux de Paris, Paris, France; 2 Equipe IMI2S, Institut des Systèmes Intelligents et de Robotique, UMR 7222, Université Paris-Sorbonne, Paris, France; 3 Pôle de Psychiatrie Infanto-Juvénile, Centre Hospitalier Intercommunal Toulon-La Seyne, Toulon, France; 4 Baruch Ivcher School of Psychology, Interdisciplinary Center, Herzlia, Israel

**Keywords:** Still Face Paradigm, motherese, vocalization, speech turn taking, mother–infant interaction

## Abstract

Language has long been identified as a powerful communicative tool among humans. Yet, pre-linguistic communication, which is common in many species, is also used by human infants prior to the acquisition of language. The potential communicational value of pre-linguistic vocal interactions between human infants and mothers has been studied in the past decades. With 120 dyads (mothers and three- or six-month-old infants), we used the classical Still Face Paradigm (SFP) in which mothers interact freely with their infants, then refrain from communication (Still Face, SF), and finally resume play. We employed innovative automated techniques to measure infant and maternal vocalization and pause, and dyadic parameters (infant response to mother, joint silence and overlap) and the emotional component of Infant Directed Speech (e-IDS) throughout the interaction. We showed that: (i) during the initial free play mothers use longer vocalizations and more e-IDS when they interact with older infants and (ii) infant boys exhibit longer vocalizations and shorter pauses than girls. (iii) During the SF and reunion phases, infants show marked and sustained changes in vocalizations but their mothers do not and (iv) mother–infant dyadic parameters increase in the reunion phase. Our quantitative results show that infants, from the age of three months, actively participate to restore the interactive loop after communicative ruptures long before vocalizations show clear linguistic meaning. Thus, auditory signals provide from early in life a channel by which infants co-create interactions, enhancing the mother–infant bond.

## 1. Background

It is widely accepted that language acquisition is one of the most significant achievements of *Homo sapiens* [1] and allows complex communication skills to develop. Nevertheless, while language is not present at birth in human infants and does not develop in other animals, communication and social interactions are widely observed throughout the animal kingdom and occur well before the acquisition of formal language. These early communicative exchanges carry an important survival function, bind the young to the larger social group, and have an imprinting function on the infant's brain during sensitive periods for brain maturation [2,3].

Early interactions between human caregivers and their infants employ multiple social signals in various modalities (e.g. gaze, facial affect, touch, orientation) [4]. For instance, in the visual domain, the infant's smile in response to the mother's smile carries a well-known rewarding effect on caregivers [5]. In the touch modality, affectionate touch has been shown to reinforce caregiving and reduce infant's stress [6,7]. Olfaction, particularly pheromones that are present in the nipple, promotes infant bonding [8]. Such multimodal sensory input contributes to infant cognitive and social-emotional development [2].

Among these sensory stimuli, the auditory channel appears to be of particular importance in humans. Typical sensorimotor development allows neonates and young infants to accurately process and produce acoustic information [9]. Moreover, before the acquisition of mobility, infants turn specifically to the auditory modality to inform caregivers of their needs; infants cry from the first moment of life, using an ‘alarm call function’, and caregivers are particularly sensitive to infant crying [10–12]. The auditory route also supports a complex interactive system that precedes language acquisition, and pre-linguistic infants can produce a wide range of social signals through this modality, such as crying, babbling, vocalizing, singing, or even grunting [13]. Further, the temporal structure of the interaction, for instance, rhythmicity and synchrony in silence and vocalizations, has been studied for decades [14–18]. A recent investigation of speech turns in natural infant–mother interaction supported an active role of infants aged as early as two months during speech turns [19].

Additionally, during dyadic interaction, caregivers are sensitive to infant vocalization, and their own vocal production regulates that of the infant [20,21]. Parents may also use infant-directed speech (IDS) that conveys linguistic as well as emotional components. The emotional component of IDS (e-IDS) has specific para-linguistic characteristics [22]. Such pre-verbal ‘dialogue’ provides the foundation for the use of language to convey more complex meanings. Indeed, in Jaffe *et al*.'s pioneering work [18], several quantitative parameters of such pre-verbal dialogue also predict later attachment patterns and cognitive abilities. Thus, while the acoustic alert system is in place at birth, acoustic interactions have been shown to carry emotional and communicative value from early infancy.

One paradigm to study early interactions is the Still Face Paradigm (SFP). It is an observational paradigm developed four decades ago by Tronick and colleagues [23] and extensively used since. In this paradigm, an infant initially plays freely with a caregiver (Free Play, FP), then, the caregiver suddenly interrupts the interaction by remaining still for two minutes (Still Face, SF). Finally, the caregiver resumes play (Reunion, R). In the reunion episode, parents and infants are faced with the task of restoring previous levels of mutually positive communication, which may highlight individual differences in dyadic coordination and parental support of infants' emotion regulation [24,25]. Two studies focusing on infant vocalization have shown that during the SF episode infants' non-cry vocalization was a way to revive infant/caregiver dialogue as early as five months. They were able to distinguish two aims for this signal: one serves to signal an emotional reaction, the other functions as an interactional regulatory tool [26,27].

Studies have used the SFP to address the effects of separation on infants' physiology and behaviour, to test the consequences of early deprivation, and to measure infant attachment [23,28,29]. Similarly, the SFP has been used to tap infant stress physiology and has shown to increase infants cortisol levels [21]. However, this increase of cortisol is not systematic, and SF's stressful effects may be attenuated by using affectionate touch [7]. In summary, the SFP provides a window into the dynamics of the interaction, the change or stability in dyadic flexibility of infant–caregiver dyads, and how infants cope with communicative rupture and repair.

However, several questions remain: does infant vocalization define an adaptive ability during the SF? Can this ability be observed in the first months of life? If such ability is present, is the pattern of infant response to maternal vocalization similar to that observed in older infants? Finally, do very young infants adapt their vocalization patterns to the situation in ways that dynamically consider its influence on the mother or is such vocalization just an alarm call produced as a social response to any stressor?

To date, experimental studies using the SF have mainly relied on observational and quantitative assessments using qualitative standardized scales or micro-analytic methods (e.g. [30]). Automated systems to detect vocal productions have also been used in the past in a few studies. The most compelling study from Jaffe *et al.* [18] continuously measured vocalization and pause of each partner and defined several dyadic parameters (switching pause, non-interruptive simultaneous speech and interruptive simultaneous speech). Then reciprocity between partners was measured using time-series analysis to calculate a global coefficient of synchrony, namely coordinated interpersonal timing [31].

Recent development in social signal processing methods, based on technological advances, now allows novel quantitative approaches to study interaction dynamics more precisely and includes the use of a specific algorithm to separate the emotional component of IDS [21]. The present study addresses the issue of how vocal interaction in early infancy might be used by the infant to re-establish communication following the experimental rupture of the SF, above and beyond its mere alert function. To this end, we developed an experimental setting in which we used: (i) the Still Face Paradigm; (ii) two infant age groups: three- and six-month-old infants; (iii) three SF conditions to provide a gradient of stress; (iv) computational analysis of audio mother–infant interaction including automatic measures of both low level features (vocalization and pause of each partner, and dyadic parameters such as overlap, joint silence, and infant response to maternal vocalization) [32] and high level features, namely mothers' emotional speech (e-IDS) [22,33].

## 2. Method

### 2.1. Participants

We included 121 dyads in total, corresponding to 20 dyads per experiment according to age (three months versus six months) and SF subtypes (SF + touch versus classic SF versus SF + arm-restraint). Each dyad was randomized to one experimental condition. Of the infants, 68 (56%) were three months and 53 (44%) were six months; 64 (53%) were female and 57 (47%) were male. Mean age of mothers of three-month infants was 29.59 years (±4.42), mean age of six-month infants was 28.72 years (±4.82) with no significant difference between the groups. Exclusion criteria included premature birth, birth-related complications, and illness. The research was approved by the Institutional Review Board (Bar-Ilan University) and conducted according to ethical standards, and all participants signed an informed consent. After randomization, 32 (26%) dyads were allocated to SF + affectionate touch, 41 (34%) were allocated to SF + arm-restraint and 48 (40%) were allocated to classic SF.

### 2.2. Still Face Procedure

**Figure 1.**
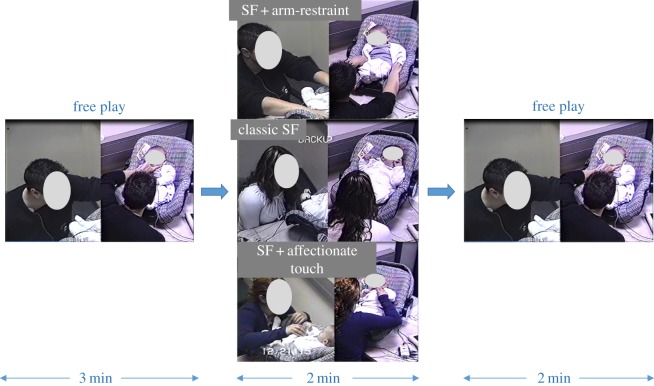
Still face experimental protocol and its gradient of stress. In the current experiment, we tested three- and six-month-old infants using the still face (SF) paradigm. To create a gradient of stress, we refined the SF paradigm by introducing two new conditions using motor cues besides the classic SF: first a condition called SF + affectionate touch (bottom) was performed with mothers asked to tenderly touch their infant during SF; second a condition called SF + arm-restraint (top) with mothers asked to actively block their infant's arms on the baby chair during SF.

After arriving at the laboratory with the mother, the infant was seated in an infant-seat mounted on a table and the mother sat facing him/her. Mothers were instructed to play freely with the infant for 3 min (FP), to maintain still face with or without affectionate touch or arm-restraint for 2 min (SF), and resume play for an additional 2 min (R). Interactions were videotaped for later coding from a control room using two cameras placed on adjacent walls and a split-screen video mixer (Flip Mino HD digital camcorder—Cisco, Irvine, CA, USA). A tap on the window signalled the time to move to the next episode of the paradigm. Compared to the classical SFP that starts with a 2 min free play, we increased from 2 to 3 min the first period of free play to ensure a cortisol response [7] (figure 1).

Thirty-two dyads were randomly selected to the SF + affectionate touch (SF+T) condition, 48 to the classic SF condition and 41 to the SF + arm-restraint (SF+A) condition. No instructions as to tactile contact during free play were provided. During the SF, mothers in the classical condition (SF) were asked to maintain a still face and refrain from any tactile, vocal, or affective communication. Mothers in the touch condition (SF+T) were instructed to keep tactile contact with the child in whichever way they chose while maintaining a still face and refraining from all affective or vocal communication. Mothers in the arm-restraint condition (SF+A) were asked to actively block their infant's arms on the seat during the SF, maintain a still face and refrain from any kind of communication.

### 2.3. Analysis of mother–infant low level audio features

**Figure 2.**
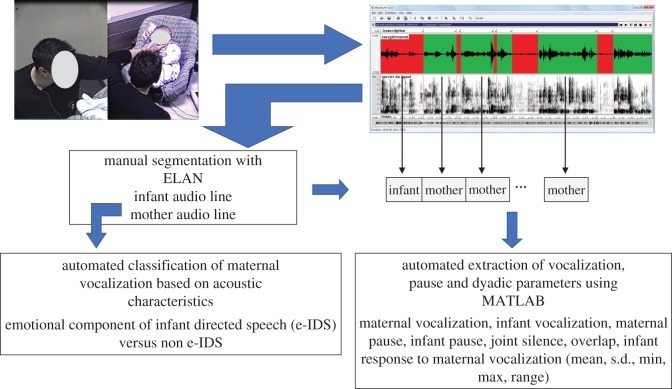
Feature extraction based on the audio line recorded during the still face experimental protocol. From the audio line, we first segmented and annotated the speakers' vocalization to separate the infant and the mother audio lines. Then we extracted maternal vocalization, infant vocalization, maternal pause, infant pause, joint silence, overlap and infant response to maternal vocalization using an automated algorithm. Finally, we classified maternal vocalization based on the presence of the emotional component of infant directed speech.

Turn-taking refers to the process by which people involved in a conversation decide who is to speak next. In this context, speech turn-taking stands for a set of quantitative parameters that capture the dynamics of the conversation (defined below). To assess this set of parameters, during mother–infant interaction we followed the methods developed by Weisman *et al.* [21]. This requires three different steps: (i) manual segmentation; (ii) annotation; (iii) extraction of targeted features (figure 2). In this study, we used a linguistic annotation tool called ELAN to segment the speakers' vocalization and annotate each vocalization in terms of the dialogue acts and speakers. The infant's and mother's utterances were labelled by two annotators (blind to SF and conditions) as vocalization (including laugh, singing and crying) or other noise. We obtained 35 843 segments of vocalization (21 722 from mothers and 14 121 from infants). Duration of the segments ranged from 0.1 to 39 s. Mothers' audio interactions were classified as: maternal vocalization (meaningful vocalizations, laugh, singing, animals' sound) or other noise (clap the hand, snap fingers or snap the tongue, mouth's noise…). Similarly, infants' audio production was defined as: infant vocalization (babbling vocalizations, laugh and cry) or other noise. Cohen's kappa between the two annotators was calculated for each dyad, each task and each item of the grid. For all items, the kappa values were between 0.82 and 1.

From the annotation, we extracted all the speech turns of the infant and the mother. A speech turn is a continuous stream of speech with less than 150 ms of silence. We obtained a list of triples: speaker label (infant or mother), start time, duration of speech turn. From these triples, we also deduced the start time and duration of the time segments when the mother and/or the infant were not speaking (pauses). Therefore, we extracted *Maternal Vocalizations* (the durations of the speaking segments for the mother); *Infant Vocalizations* (the durations of the speaking segments for the infant); *Maternal Pauses* (the durations of the silence segments for the mother, a silence being defined by greater than 150 ms between 2 mother speech slots); *Infant Pauses* (the durations of the silence segments for the infant, a silence being defined by greater than 150 ms between 2 infant speech slots).

We also extracted three features involving simultaneously both interactive partners, so-called dyadic variables in table 2 and electronic supplementary material, tables S1 and S2: (i) *Joint Silence* was defined as sequences of time during which neither participant was speaking for more than 150 ms. Joint silence may reflect a rupture of speech synchrony or a lack of speech interaction between the participants. (ii) *Overlap Ratio* was defined as duration of vocalization overlaps between mothers and infants divided by the duration of the total interaction. It measures the proportion of interactional time in which both participants were vocalizing simultaneously. (iii) *Infant Response to Maternal Vocalization Ratio* was the number of infant's responses to his/her maternal vocalization within a time limit of 3 s divided by the number of maternal vocalizations during the time paradigm. The 3 s window was based on available literature on early mother–infant interaction [4,34–39]. This feature estimated the ratio of time-related responses by the infant to his/her mother's vocal features. By definition, mothers remain still during SF, so that defining mother speech parameters during this time is inappropriate. However, the infant may vocalize, so we applied the same annotation for each infant during SF and extracted his/her vocalization.

### 2.4. Affective speech analysis of the mother (high level audio features)

Previous work has shown that the nature of the parental vocalization may also be of importance and may shape parent–infant interaction. In particular, ‘IDS’ refers to the spontaneous way in which mothers, fathers and caregivers speak with infants and young children [22]. The emotional component of IDS (e-IDS) has specific acoustic characteristics: higher mean pitch, wider pitch range, low speaking rate and vowel hyper-articulation [22]. Whereas the ‘manual’ analysis of acoustic components of the voice takes a very long time and only allows for the study of very short voice segments, the use of an automatic classifier made it possible to conduct an extensive study of all maternal vocalizations based on their acoustic characteristics. The segments of maternal vocalizations were analysed using a computerized classifier for categorization as ‘e-IDS’ or ‘non e-IDS/other speech’ initially developed to analyse home movies (HM) recorded in Italian [20] (figure 2).

Most commonly, features employed in the design of computerized classifier aim to capture pitch, duration, energy of vocalizations as well as global dynamics of spectrum. These are traditionally respectively implemented as statistics of fundamental frequency, duration and energy of vocalizations [32,40,41]. These features are usually termed as supra-segmental. Mel frequency cepstral coefficients (MFCC) capture short-term dynamics of spectrum and are termed segmental features. The system exploits the combination of two classifiers, segmental and supra-segmental, that are weighted and fused to reach best classification rates [33]. Consequently, the utterances were characterized by both segmental (MFCC) and supra-segmental/prosodics (e.g. statistics with regard to fundamental frequency (F0 or pitch), energy and duration) features. We tested both GMM (Gaussian mixture model) and k-NN (k-nearest neighbours) classifiers and different weight for fusion of segmental and supra-segmental classification. In its most effective configuration, the detector used only the GMM classifier for both segmental and supra-segmental features (*M*, number of Gaussians for the GMM classifier: *M* = 12 and *M* = 15, respectively, and *λ* = weighting coefficient used in the equation fusion: *λ* = 0.4). For the purpose of the current study, we explored the performance of our e-IDS classifier in Israeli mothers. We analysed 200 sequences from Israeli mothers (100 e-IDS versus 100 non e-IDS) that were blindly validated by two psycholinguists. The system's performance was: accuracy = 88.2% (95% CI: 82.4–92.2%); PPV = 76.5% (95% CI: 70.6–82.4%). This level of prediction made it suitable for further analysis of the entire dataset.

Based on this automatic detection of e-IDS, we created two sub-classes for maternal vocalization: e-IDS versus non e-IDS. Two variables were derived: *e-IDS Ratio* (duration of IDS vocalization/duration of interaction) and *non e-IDS Ratio* (duration of non e-IDS vocalization/duration of interaction). We also derived two *Infant response to maternal vocalization* ratios: *Infant response to maternal vocalization e-IDS Ratio*, *Infant response to maternal vocalization non e-IDS Ratio* which reflect the ratio of time during which the infant vocalizes in response to his/her mother's e-IDS and non e-IDS).

### 2.5. Statistical analysis

Statistical analyses were performed using R software, v. 2.12.2. We used linear mixed models (LMM) applied on means of mother/infant/dyadic parameters (taken as the response variable of the model) to assess the effect of the following parameters (taken as the explanatory variables): (i) age (three months or six months); (ii) type of SF (touch versus classic and arm-restraint versus classic); (iii) gender (male versus female); and (iv) time (before, during and after SF). For each response variable, the normal distribution was checked. In the case of variables that did not show normal distributions, a logarithmic transformation was necessary before processing the LMM. For every test, the level of significance alpha was fixed at 5%.

To assess infant vocalization change during the three study periods (before SF, during SF and after SF) a secondary analysis using the same LMM was used with a change in the time variable that became before, during and after SF. In the case of significant results, *post hoc* analyses were conducted to compare infant vocalization change by contiguous segment of time. Also, to assess effect of age and gender on free play interaction, a LMM was applied on means of mother/infant/dyadic variables during the first period of free play.

## 3. Results

### 3.1. Maternal, infant and dyadic parameters during free play

Since the first part of all experimental conditions was similar, we first describe low audio feature parameters and IDS variables during mother/infant interactions during the free play phase before SF according to age and gender (table 1). This gives us an insight into the interaction recorded in an experimental context through an unconstrained situation. The LMM applied to each variable showed that the infants' age had an effect on mother parameters only, where mothers showed longer vocalizations (*β* = 0.077, *p* = 0.022) and used more e-IDS with the six-month-old infants. Gender had an effect on both infant and dyadic parameters. Boys showed significantly longer vocalizations (*β* = 0.129, *p* =  0.008) and shorter pauses (*β* = −0.622, *p* = 0.044) than girls. Interactions involving boys showed higher response to mother rates, after either e-IDS (*β* = 0.162, *p* < 0.001) or non e-IDS (*β* =  0.091, *p* = 0.02).

**Table 1.** Speech variables [mean(s.d.)] during the free play phase according to age and gender. s.d. = standard deviation; e-IDS = emotional component of infant directed speech; SF = still face.

	three months		six months	
	girl	boy	girl	boy
mother parameters				
maternal vocalization	1.34 (0.49)	1.38 (0.49)	1.65 (0.66)	1.51 (0.46)
maternal pause	1.01 (0.30)	0.92 (0.21)	0.95 (0.21)	0.93 (0.27)
e-IDS ratio	0.22 (0.18)	0.26 (0.17)	0.30 (0.15)	0.32 (0.22)
infant parameters				
infant vocalization	0.47 (0.18)	0.62 (0.33)	0.50 (0.25)	0.60 (0.25)
infant pause	1.27 (1.15)	1.16 (1.08)	2.45 (2.61)	1.08 (0.75)
dyadic parameters				
silence	0.42 (0.13)	0.39 (0.12)	0.40 (0.10)	0.37 (0.13)
overlap	0.05 (0.05)	0.06 (0.07)	0.03 (0.04)	0.07 (0.07)
infant response to mother	0.43 (0.20)	0.49 (0.20)	0.34 (0.17)	0.50 (0.23)
infant response to mother e-IDS	0.33 (0.25)	0.46 (0.23)	0.30 (0.19)	0.52 (0.24)

### 3.2. Maternal, infant and dyadic parameters dynamics after Still Face

Using a generalized linear model to take into account simultaneously the different explanatory variables, we found that the type of SF had no influence on individual parameters (infant or mother) and little influence on dyadic parameters. There were more infant responses in the 3 s time window after a maternal vocalization (specifically e-IDS) after SF with affectionate touch than after classic SF (more infant response to mother ratio: *β* = 0.1, *p* = 0.04, and more infant response to mother e-IDS ratio: *β* = 0.13, *p* = 0.01). There were no differences between arm-restraint SF and classic SF on any parameters. Detailed data are available in the electronic supplementary material, tables S1–S3. For ease of presentation, we will group all the SFs in the rest of the manuscript. Speech variables according to time (before SF = Free Play versus after SF = Reunion) and age (three months versus six months) are given in table 2. However, there were several significant effects of age and gender, and almost all speech parameters were affected by time.

**Table 2.** Vocalization, pause and dyadic variables [mean (s.d.)] before and after the still face: effect of age, gender and time. s.d. = standard deviation; e-IDS = emotional component of infant directed speech; SF = still face.

	three months		six months		effect of		
	before SF	after SF	before SF	after SF	age	gender	time
maternal parameters							
maternal vocalization	1.36 (0.48)	1.37 (0.46)	1.59 (0.58)	1.64 (0.64)	*p* = 0.005	*p* = 0.86	*p* = 0.53
maternal pause	0.96 (0.26)	0.93 (0.40)	0.94 (0.23)	0.78 (0.27)	*p* = 0.07	*p* = 0.13	*p* = 0.003
e-IDS ratio	0.24 (0.17)	0.24 (0.18)	0.31 (0.18)	0.33 (0.22)	*p* = 0.01	*p* = 0.32	*p* = 0.34
infant parameters							
infant vocalization	0.54 (0.28)	1.18 (1.96)	0.54 (0.25)	1.17 (1.50)	*p* *=* 0.44	*p* *=* 0.008	*p* *<* 0.001
infant pause	1.22 (1.10)	0.74 (1.18)	1.88 (2.15)	0.70 (0.44)	*p* *=* 0.078	*p* *=* 0.012	*p* *<* 0.001
dyadic parameters							
silence	0.41 (0.12)	0.32 (0.15)	0.39 (0.11)	0.26 (0.12)	*p* *=* 0.035	*p* *=* 0.07	*p* *<* 0.001
overlap	0.06 (0.06)	0.13 (0.14)	0.05 (0.05)	0.14 (0.13)	*p* *=* 0.99	*p* *=* 0.02	*p* *<* 0.001
infant response to mother vocalization Ratio	0.46 (0.20)	0.59 (0.27)	0.41 (0.21)	0.58 (0.26)	*p* *=* 0.44	*p* *=* 0.009	*p* *<* 0.001
infant response to mother e-IDS vocalization Ratio	0.40 (0.25)	0.54 (0.31)	0.39 (0.24)	0.56 (0.30)	*p* *=* 0.73	*p* *=* 0.003	*p* *<* 0.001
infant response to mother non e-IDS vocalization Ratio	0.49 (0.19)	0.61 (0.27)	0.42 (0.23)	0.61 (0.29)	*p* *=* 0.66	*p* *=* 0.01	*p* *<* 0.001

Effect of age was significant mainly for some mother parameters. There was longer maternal vocalization (*β* = 0.09, *p* = 0.05) and more use of e-IDS (*β* = 0.028, *p* = 0.01) when infants were aged six months than three months. Accordingly, joint silence (*β* = −0.015, *p* = 0.035) was less frequent in interaction involving six-month-olds than three-month-olds. Effect of gender was significant mainly for infant parameters and dyadic variables. Infant vocalizations (*β* = 0.27, *p* = 0.008) were longer and infant pauses (*β* = −0.29, *p* = 0.012) were shorter in boys than girls. Also, infant responses after a maternal vocalization (both e-IDS: *β* = 0.12, *p* = 0.003 and non e-IDS: *β* = 0.10, *p* = 0.01) and, as a consequence, overlap ratio (*β* = 0.52, *p* = 0.02) were increased in boys compared to girls. Of note, mother parameters were not affected by gender.

In contrast, effect of time (meaning: Reunion versus Free Play) was significant for many parameters, independently of age, type of SF and gender (given the multivariate model analysis). On the one hand, there was an increase in length of infant vocalization and a decrease in infant pause after SF during the reunion phase. On the other, there was a decrease in maternal pause and an increase in non e-IDS ratio after SF. As a consequence all dyadic parameters changed accordingly: joint silence ratio decreased during reunion after SF whereas overlap ratio, infant responses after a maternal vocalization after both e-IDS and non e-IDS speech increased after SF.

### 3.3. Infant's speech parameters during Still Face

**Figure 3.**
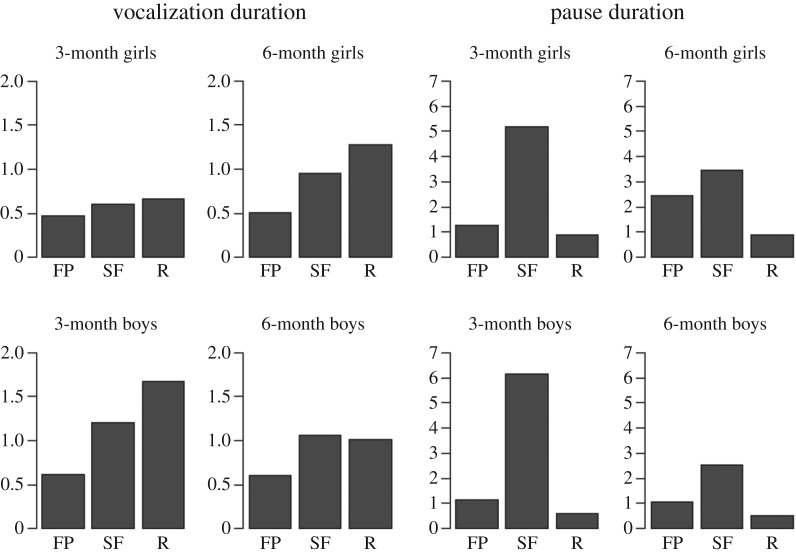
Infant vocalization and pause during the three phases of the Still Face Paradigm, according to age and gender. For all categories, length of vocalization increases during the SF when compared to FP, and the increase is sustained during Reunion after SF, except for six-month boys. Also, the length of pauses also increases during SF for all infant categories. The variation in pause length is more marked in three-month infants than in six-month infants.

Secondary analyses were performed to investigate infant's vocalization during Still Face. The effects of age, gender, type of SF, and time were entered into the models. There was no significant effect either of age or type of SF. We found a significant effect of gender, with boys showing more increase in vocalization than girls (*β* = 0.27; *p* *=* 0.01). Symmetrically, boys showed a decrease in pause compared to girls (*β* = −0.24; *p* = 0.02). Also, there was a significant effect of time, with an increase of vocalization duration that occurred during SF (*β* = 0.38; *p* < 0.001, when comparing before SF and during SF; *β* = 0.44; *p* < 0.001, when comparing before SF and after SF). There was also an increase of pause duration that occurred only during the SF (*β* = 1.02; *p* < 0.001, when comparing before SF and during SF; *β* = 1.61; *p* < 0.001, when comparing during SF and after SF) (figure 3).

During SF, in the absence of mother response, infants showed longer durations of vocalization and pauses than during FP, whatever the age and type of SF. After SF, during Reunion, infant vocalizations were even longer, but durations of pauses were shorter. In every case, the mean duration of infant pauses was shorter in Reunion than during initial Free Play. Thus, infants adapted to the presence or absence of interaction, and after a break in the interaction, they sustained the come-back of the interaction by being more active in the interaction than they were during the initial free play: vocalizations were longer than before SF, and pauses become shorter than during, and before SF.

## 4. Discussion

**Figure 4.**
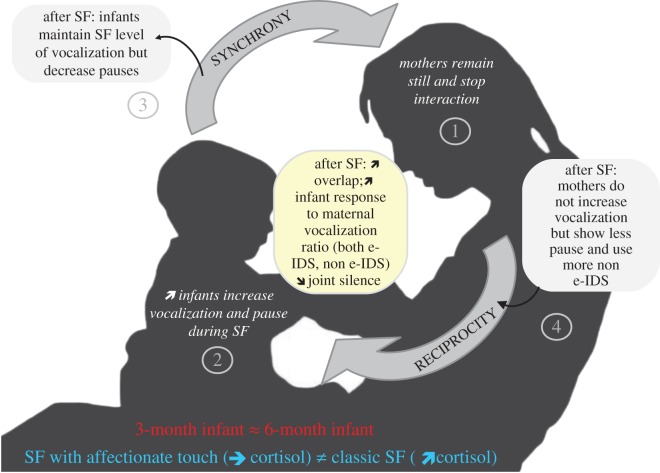
Impact of mother still face on interaction dynamics in terms of vocalization in infants aged three and six months. In this figure, we summarize the effect of mother still face (SF) on interaction dynamics in terms of vocalization. Mothers remain still and stop interaction. Infants during the SF phase increase their vocalization and pause duration. When interaction restarts after SF, infants continue to keep a high level of vocalization duration which impacts the dyadic parameters. After SF, joint silences decrease, overlap ratio increases as well as infant response to maternal vocalization, after both e-IDS and non e-IDS. e-IDS = emotional component of Infant Directed Speech. Joint Silence = sequence of time during which none of the participant is speaking for more than 150 ms. Overlap ratio = the percentage of interactional time when both mother and infant are talking at the same time. Infant response to maternal vocalization ratio = the number of infant's response to its maternal vocalization within a time limit of 3 s divided by the number of maternal vocalization during the time paradigm.

Using the Still Face Paradigm with a large sample of mother–infant dyads and novel computational techniques, we observed a consistent pattern of infant reactivity to the stressful SF paradigm. The features of the interaction dynamics are summarized in figure 4. In all experimental conditions, for both ages and genders, infant vocalization duration increased significantly during the SF. Following the interactive rupture, during the reunion phase, infant vocalization further increased or remained the same as during the SF. In contrast, infant pause decreased below the level observed in the initial free play and this was found again in all groups, indicating that infants are adapting to caregiver return. These results are in accordance with previous observations on infants' behaviour during the SF in other modalities [30,42], showing that infants attempt to (i) maintain the interaction through actions such as sustained gaze towards the caregiver or increased babbling, and (ii) control their emotional arousal via behaviours such as turning attention away from parent or self-comforting behaviour. During the reunion phase, dyadic parameters changed in parallel; the joint silence decreased, the overlap increased, and, most importantly, infant response to maternal vocalization after both e-IDS and non e-IDS increased as well. Thus, the increase in vocalization duration appears to be an infant initiated mechanism observed as early as three months of age to elicit maternal support following absence and repair communication ruptures.

### 4.1. Interpreting the communicative value of early vocalization length

Interestingly, in this experiment, we found no major effect of the type of SF on speech features. In a previous study, using a similar paradigm, associated with hormonal and neurophysiological measures, Feldman *et al*. [7] showed that SF with affectionate touch was associated with diminished stress response when compared to classic SF: cortisol reactivity was higher in infants exposed to the classic SF condition. Furthermore, in that study, while cortisol decreased during the reunion phase for infants in the SF with affectionate touch, it further increased for those in the classic condition. Vagal tone also showed a greater decrease in classic SF [7]. In another study, it was shown that SF with arm-restraint elicited a frustration response starting at two months [43]. Given that (i) the current experiment used different SF conditions related to various intensities of stress elicitation; and (ii) the type of SF had no influence on infant behaviour, the pattern of infant responses cannot be solely regarded as a response to stress. Indeed, while the level of stress may differ between the three conditions, it does not affect the pattern of infant vocalizations during and after SF. In other words, in this experiment, the SF may not be only used and interpreted as an early stress paradigm, but as a sudden break in the interaction.

One other striking result is the absence of significant quantitative differences of behavioural response between infants aged three and six months. To date, research in early interaction has mainly focused on older infants, who were thought to show dyadic patterns of behaviour mainly around and after six months of age [44]. Here, the only significant difference between age groups was found in mothers' vocalizations: mothers used longer vocalization with their six-month-old infants and used the emotional component of IDS more. This means that from the mother's standpoint, mothers discriminate spontaneously between the two age groups. However, our results clearly show that infants aged three months show the same patterns in terms of vocalization, silence and response to maternal vocalization through the three phases of the SF, regardless of the SF type. These results are in line with Rochat *et al.* [45] who showed that from the age of two months, at a time of development when inter-subjectivity begins to emerge, infants show sensitivity to the timing and structure of the interaction. Finally, gender revealed several significant effects from the infants' perspective while there was no difference in mothers' speech parameters by infant gender. These results, showing longer vocalizations, shorter pauses, and more overlap in boys, regardless of age, have, to our knowledge, not been found in earlier studies with infant aged three months. For six-month infants, Weinberg *et al*. [10] reported a multimodal analysis of infant–mother interaction during a classic SF experiment. They found that boys had more neutral--positive vocalization than girls during all periods of the SF experiment. Both boys and girls increased fussy vocalization and crying during SF. These findings possibly indicate more spontaneous early social activity and more reactivity to social rupture in boys compared to girls. Future research is required to test whether this may relate to boys' greater sensitivity to early stress (e.g. [10,46]).

A recent study using recordings of mothers and infants aged two to five months in naturalistic contexts reported similar results [19]. They showed that from an early age, infants are taking part in the speech turn not only in a structured way but also in an active manner, which improves over the first months of life. From a developmental perspective, these cumulative findings indicate that as early as three months of age infants play a significant and active role in eliciting and then sustaining the caregivers' response using vocalization in the SF paradigm. Our specific contribution to this field of study is methodological through the use of quantitative measures that provided several benefits. First, it makes it possible to analyse a large sample. Second, it enables the continuous measurement of infant response to maternal vocalization and also the use of e-IDS. Even if we detailed only one way of the interaction (that is infant response to maternal vocalization), our results support the previous findings and the concept of co-regulation of communication in dyads that has been studied for decades [47–49]. Our findings also confirm the very few studies that use automatic methods and mathematical models to capture reciprocity during early vocal interactions in natural settings [18,19]. Current studies also include the automated measures of other social signals (e.g. gaze, smile, and head movements [50] or body movements [51]) to model interactions through multimodality.

In the same vein, within the social-interaction literature there is often the assumption that the parent plays the major adaptive role in response to stereotypical infant signals. We suggest that, in the context of the SFP, infant communication signals are not just responses to stress stimuli, and their communication, as described by Dennett, is marked by intentionality [52] (table 3). Infant reactivity plays an active role in re-establishing the interaction during the reunion phase, and this is seen in both the high level (e-IDS) and low level features (infant vocalization and infant response to maternal vocalization), as well as in the dynamics of the interaction, rather than on the discrete responses of each member of the dyad. Several studies have shown that synchrony is a social signal in itself and a key feature of early interaction in dyads (for a review see [51]).

**Table 3.** Hierarchy of intentional communication (from [52]). TOM = Theory of mind; SF = Still Face; e-IDS = emotional component of Infant Directed Speech.

level	definition	present in animals	infant
first order	Alarm calls asking for protection or promoting protection of others	Ultrasounds in mouse [53] Vocalization in velvet monkeys [54]	At both three and six months, infants express distress by increasing vocalization during and after SF
second order	Communication to others showing variation with social context, or with specific partners	Audience effect in vocalization production of velvet monkeys [55,56] Gesture communication in chimpanzees that adapt to attentive (silent gesture) and inattentive (touching gesture) partners Vocalization and speech turn tacking in marmoset Monkeys [57]	At both three and six months, infants' increase of vocalization is not only dependent on stress Infant vocalization after SF is independent of e-IDS so is not dependent on the other partner's vocalization
third order	Communication aiming at making listeners believe that the speaker wants them to do something (imperative request)	Some non-human primates have the ability to attribute knowledge to congeners (e.g. [58])	Children around 2 years understand that partners may have knowledge and thoughts but cannot attribute false belief (first order of TOM)
fourth order	Communication aiming at making listeners believe that the speaker wants them to believe that he wants them to do something (declarative request)	Not found in the animal world	Children around 4 years develop the ability to attribute false-belief (second order of TOM) [59]

Consistent with Feldman [60], we suggest that these less visible parameters of the interaction contribute to the envelope of ‘low intensity trophallaxis’ that allows bonding in mammals. Trophallaxis is defined as the reciprocal multi-sensory stimulation of low intensity that elicits approach response. In this work, new features of the interaction appear salient when quantitative tools are used for each partner and both partners together (dyadic parameters). Thus, apart from the nature of acoustic production, the length and rhythm of vocalizations, pauses and infant response to maternal vocalization may constitute a distinct coded signature of the mother–infant bond that unfolds during their social interaction [18].

### 4.2. Interpreting intentionality and infant vocalization within an evolutionary perspective

Animal communication and social interaction is based on distinct signalling channels and sensory modalities. Depending on species, studies have depicted a variety of social cues involved in bonding and affiliative behaviour related to both the source (infant versus caregiver) and the modality (e.g. auditory, visual) of communication [53]. As to acoustic stimuli, signals are expressed in a variety of ways, as are the goals of interaction through this channel. For examples, rodent pups' separation distress is expressed through ultrasonic vocalizations [53]. Alarm calls seeking protection or promoting protection of others exist in velvet monkeys employing different types of vocalizations depending on the threat [54]. In bottlenose dolphins, the signature whistle is specific for each individual and is used as a recognition signal with peers. Such individually tailored signals are transmitted from mother to offspring via a process of acoustic imprinting [3]. In adult non-human primates, more complex forms of communication involving vocalizations that encode simultaneously for semantic and emotional information have been reported and are specific to social context and interactive partner [55,56].

Turn-taking in conversation is a common feature in various human cultures. From the current findings and the few other studies conducted in natural settings that included very young infants [18,19], it appears to be present very early in development and long before infant vocalization can be recognized as linguistic. This raises questions about its biological basis and evolutionary trajectory. Functional convergence is a widespread phenomenon in evolution, revealing sometimes striking functional similarities between very distant species. In a variety of social species, vocal exchange can be found. This is the case in dolphins [3], elephants [11], monkeys [61] and birds [62,42]. It appears that in many species temporal and structural regularities in vocal interactions are shaped and depend on the species' social structure. From the detailed review of Henry *et al*. [63], we want to emphasize some important implications in terms of human bonding. First, in the European starling (i) vocal interactions in several animal groups are clearly regulated, especially in terms of timing; (ii) both intra- and inter-specific variations are observed that hint at possible evolutionary processes and implications for bonding; more overlap and communal chorusing in tight social groups, more alternation between distant neighbours, with sometimes both types of exchanges in the same species according to context; (iii) (male) starlings take into account the social context when they are singing. By increasing interval duration between two whistles, they clearly leave space for other birds to reply and therefore make turn-taking possible. Second, animal studies converge to show that turn-taking in species may be acquired during development and shaped by adult adaptation to the young [64–66]. In the case of marmoset monkeys, Takahashi and colleagues, using computational methods similar to ours, recently investigated early development of infants' vocalization and turn-taking with parents [57]. Infants gradually engage in vocal turn-taking during the first month of life but parents are stable in their behaviour. Considering that marmoset monkeys develop 12 times faster than humans, the infants undergo the same trajectory of change for vocal turn-taking as humans, and do so during the same life-history stage. Hence, the biological basis of this process, widespread in animals, remains unclear, as do the evolutionary pathways.

## 5. Conclusion

In this study, we used (i) a traditional SF paradigm for the study of early interactions and communication rupture between mothers and their infants aged three and six months, (ii) coupled with a novel methodological approach based on quantitative assessments of the dynamics of vocal interactions. We show that (i) as early as three months of age, infants use vocalization to restore interactive ruptures and follow communication rules; (ii) this phenomenon cannot be explained by simple stress reactivity; (iii) this active infant vocal behaviour represents an infant-initiated mechanism that is not a response to changes in maternal vocalization (e.g. by using more e-IDS) after the interactive rupture. Taken together, our findings underscore the crucial and active role of the infant in regulating the interactional loop starting at an early age. This early communicative ability may also contribute to bonding.

## Supplementary Material

bourvis_ESM_1.pdf

## Supplementary Material

bourvis_ESM_2.pdf

## Supplementary Material

bourvis_ESM_3.pdf

## Data accessibility

Supporting data have been deposited in external repositories. The raw data are summarized in an excel file on Dryad: http://dx.doi.org/10.5061/dryad.61668 [67]. Tools used to assess speech turn can be downloaded on https://github.com/syncpy/SyncPy, an open-source library developed by our laboratory.
