# Supplementary material for: Pre-linguistic infants employ complex communicative loops to engage mothers in social exchanges and repair interaction ruptures
Source: R Soc Open Sci. 2018 Jan 24;5(1):170274. doi: 10.1098/rsos.170274 (PMC5792867; doi:10.1098/rsos.170274)
Supplement: bourvis_ESM_1.pdf [file rsos170274supp1.pdf]

**Table S1. Vocalization, pause and dyadic variables during mother / 3-month infant interaction before and after still face according to each SF condition (touching *vs.* classic *vs.* arm-restraint)**			
	**Before still face**		
	With Touching	Classic	With arms' blocking
Mother parameters			
Vocalization: Mean (SD)	1.38 (0.63)	1.38 (0.52)	1.31 (0.32)
Pause: Mean (SD)	0.89 (0.2)	1 (0.5)	0.96 (0.3)
Motherese ratio: Mean (SD)	0.2 (0.16)	0.23 (0.21)	0.27 (0.13)
Non Motherese ratio: Mean (SD)	0.33 (0.16)	0.29 (0.15)	0.26 (0.14)
Infant parameters			
Vocalization: Mean (SD)	0.48 (0.17)	0.57 (0.33)	0.55 (0.27)
Pause: Mean (SD)	1.64 (1.53)	0.95 (0.8)	1.25 (1.02)
Dyadic parameters			
Joint Silence Ratio: Mean (SD)	0.4 (0.12)	0.42 (0.12)	0.4 (0.13)
Overlap Ratio: Mean (SD)	0.06 (0.06)	0.04 (0.06)	0.06 (0.07)
Infant response to maternal vocalization Ratio: Mean (SD)	0.56 (0.21)	0.38 (0.2)	0.5 (0.16)
Infant response to maternal vocalization Ratio > eIDS: Mean (SD)	0.52 (0.29)	0.28 (0.22)	0.46 (0.18)
Infant response to maternal vocalization Ratio > Non-eIDS: Mean (SD)	0.56 (0.2)	0.41 (0.21)	0.51 (0.17)
	**After still face**		
	With Touching	Classic	With arms' blocking
Mother parameters			
Vocalization: Mean (SD)	1.34 (0.45)	1.29 (0.28)	1.49 (0.62)
Pause: Mean (SD)	0.84 (0.24)	1.05 (0.47)	0.83 (0.37)
Motherese ratio: Mean (SD)	0.21 (0.21)	0.21 (0.18)	0.3 (0.16)
Non Motherese ratio: Mean (SD)	0.36 (0.18)	0.31 (0.15)	0.29 (0.2)
Infant parameters			
Vocalization: Mean (SD)	0.79 (0.61)	1.05 (0.97)	1.61 (3.16)
Pause: Mean (SD)	0.6 (0.63)	1.04 (1.72)	0.44 (0.13)
Dyadic parameters			
Joint Silence: Mean (SD)	0.3 (0.15)	0.36 (0.13)	0.28 (0.17)
Overlap Ratio: Mean (SD)	0.14 (0.13)	0.11 (0.11)	0.15 (0.17)
Infant response to maternal vocalization Ratio: Mean (SD)	0.67 (0.24)	0.53 (0.28)	0.6 (0.29)
Infant response to maternal vocalization Ratio > eIDS: Mean (SD)	0.59 (0.37)	0.47 (0.26)	0.56 (0.31)
Infant response to maternal vocalization Ratio > Non-eIDS: Mean (SD)	0.7 (0.24)	0.53 (0.29)	0.61 (0.29)
