# Supplementary material for: Pre-linguistic infants employ complex communicative loops to engage mothers in social exchanges and repair interaction ruptures
Source: R Soc Open Sci. 2018 Jan 24;5(1):170274. doi: 10.1098/rsos.170274 (PMC5792867; doi:10.1098/rsos.170274)
Supplement: bourvis_ESM_2.pdf [file rsos170274supp2.pdf]

**Table S2. Vocalization, pause and dyadic variables during mother / 6-month infant interaction before and after still face according to each SF condition (touching *vs.* classic *vs.* arm-restraint)**			
	BEFORE STILL FACE		
	With Touching	Classic	With arms' blocking
Mother parameters			
Vocalization: Mean (SD)	1.58 (0.65)	1.68 (0.69)	1.5 (0.38)
Pause: Mean (SD)	0.99 (0.2)	0.91 (0.21)	0.94 (0.28)
Motherese ratio: Mean (SD)	0.31 (0.18)	0.33 (0.19)	0.29 (0.18)
Non Motherese ratio: Mean (SD)	0.24 (0.12)	0.26 (0.17)	0.24 (0.14)
Infant parameters			
Vocalization: Mean (SD)	0.55 (0.2)	0.54 (0.3)	0.54 (0.26)
Pause: Mean (SD)	1.89 (2.49)	1.22 (1.08)	2.39 (2.45)
Dyadic parameters			
Joint Silence Ratio: Mean (SD)	0.4 (0.14)	0.36 (0.09)	0.4 (0.11)
Overlap Ratio: Mean (SD)	0.04 (0.05)	0.05 (0.06)	0.05 (0.06)
Infant response to maternal vocalization Ratio: Mean (SD)	0.37 (0.18)	0.4 (0.2)	0.45 (0.25)
Infant response to maternal vocalization Ratio > eIDS: Mean (SD)	0.38 (0.23)	0.35 (0.21)	0.44 (0.27)
Infant response to maternal vocalization Ratio > Non-eIDS: Mean (SD)	0.37 (0.17)	0.43 (0.23)	0.46 (0.38)
	AFTER STILL FACE		
	With Touching	Classic	With arms' blocking
Mother parameters			
Vocalization: Mean (SD)	1.61 (0.78)	1.56 (0.51)	1.76 (0.65)
Pause: Mean (SD)	0.75 (0.24)	0.81 (0.33)	0.76 (0.22)
Motherese ratio: Mean (SD)	0.28 (0.21)	0.33 (0.23)	0.37 (0.22)
Non Motherese ratio: Mean (SD)	0.34 (0.2)	0.3 (0.2)	0.26 (0.17)
Infant parameters			
Vocalization: Mean (SD)	0.91 (0.47)	1.52 (2.35)	1.02 (0.76)
Pause: Mean (SD)	0.78 (0.48)	0.68 (0.42)	0.65 (0.44)
Dyadic parameters			
Joint Silence Ratio: Mean (SD)	0.28 (0.13)	0.25 (0.11)	0.25 (0.11)
Overlap Ratio: Mean (SD)	0.14 (0.13)	0.14 (0.15)	0.14 (0.13)
Infant response to maternal vocalization Ratio: Mean (SD)	0.58 (0.26)	0.56 (025)	0.59 (0.29)
Infant response to maternal vocalization Ratio > eIDS: Mean (SD)	0.58 (0.3)	0.52 (0.32)	0.59 (0.28)
Infant response to maternal vocalization Ratio > Non-eIDS: Mean (SD)	0.58 (0.27)	0.59 (0.28)	0.65 (0.31)
