# Supplementary material for: Pre-linguistic infants employ complex communicative loops to engage mothers in social exchanges and repair interaction ruptures
Source: R Soc Open Sci. 2018 Jan 24;5(1):170274. doi: 10.1098/rsos.170274 (PMC5792867; doi:10.1098/rsos.170274)
Supplement: bourvis_ESM_3.pdf [file rsos170274supp3.pdf]

**Table S3: Linear Mixed Model of vocalization, pause and dyadic variables during mother infant interaction using a still face paradigm**					
**MOTHER PARAMETERS**					
	Age effect	Type of SF effect	Gender effect	Time effect	
		Touch vs. Classic	Arm vs. Classic		
Mother Vocalization Mean	**0.09 *(p=0.005)***	0.005 *(p=0.96)*	0.04 *(p=0.67)*	0.01 *(p=0.86)*	-0.027 *(p=0.53)*
Mother Pause Mean	-0.028 *(p=0.07)*	-0.09 *(p = 0.12)*	-0.07(p=0.16)	-0.069 (p=0.13)	**0.09 (p= 0.003)**
Motherese Ratio	**0.028 (p=0.01)**	-0.018 (p =0.65)	0.034 p=0.37)	0.033 (p=0.32)	-0.010 (p=0.34)
Non Motherese Ratio	-0.011 (p=0.26)	-0.025 (p=0.50)	0.025 (p=0.45)	-0.030 (p=0.31)	**-0.038 (p<0.001)**
**INFANT PARAMETERS**					
	Age effect	Type of SF effect	Gender effect	Time effect	
		Touch vs. Classic	Arm vs. Classic	Gender effect	Time effect
Log(Infant Vocalization) Mean	0.03 (p=0.44)	-0.05 (p=0.67)	-0.04 (p=075)	**0.27 (p=0.008)**	**-0.45 (p<0.001)**
Log(Infant Pause) Mean	0.067 (p=0.078)	0.027 (p=0.85)	0.11 (p=0.39)	**-0.29 (p=0.012)**	**0.59 (p<0.001)**
**DYADIC PARAMETERS**					
	Age effect	Type of SF effect	Gender effect	Time effect	
		Touch vs. Classic	Arm vs. Classic	Gender effect	Time effect
Joint Silence Ratio Mean	**-0.015 (p=0.035)**	-0.01 (p=0.62)	-0.02 (p=0.47)	-0.04 (p=0.07)	**0.11 (p<0.001)**
Overlap Ratio Mean	0.001 (p=0.99)	0.32 (0.23)	0.16 (p=0.51)	**0.52 (p=0.02)**	**-0.86 (p<0.001)**
Infant response to maternal vocalization Ratio: Mean (SD)	-0.01 (p=0.44)	**0.1 (p=0.04)**	0.06 (p=0.14)	**0.10 (p=0.009)**	**-0.14 (p<0.001)**
Infant response to maternal vocalization Ratio > eIDS: Mean (SD)	0.005 (p=0.73)	**0.13 (p=0.01)**	0.10 (p=0.04)	**0.12 (p=0.003)**	**-0.15 (p<0.001)**
Infant response to maternal vocalization Ratio > Non-eIDS: Mean (SD)	-0.006 (p=0.66)	0.08 (p=0.09)	0.06 (p=0.17)	**0.10 (p=0.01)**	**-0.15 (p<0.001)**
